# A toolbox for conditional control of gene expression in apicomplexan parasites

**DOI:** 10.1111/mmi.14821

**Published:** 2021-10-13

**Authors:** Sylvie Briquet, Mathieu Gissot, Olivier Silvie

**Affiliations:** ^1^ INSERM CNRS Centre d’Immunologie et des Maladies Infectieuses Sorbonne Université Paris France; ^2^ CNRS Inserm CHU Lille Institut Pasteur de Lille Center for Infection and Immunity of Lille CIIL, Univ. Lille Lille France

**Keywords:** apicomplexa, conditional genome editing, protein degron systems, regulatable promoters, ribozymes

## Abstract

Apicomplexan parasites encompass diverse pathogens for humans and animals, including the causative agents of malaria and toxoplasmosis, *Plasmodium* spp. and *Toxoplasma gondii*. Genetic manipulation of these parasites has become central to explore parasite biology, unravel gene function and identify new targets for therapeutic strategies. Tremendous progress has been achieved over the past years with the advent of next generation sequencing and powerful genome editing methods. In particular, various methods for conditional gene expression have been developed in both *Plasmodium* and *Toxoplasma* to knockout or knockdown essential genes, or for inducible expression of master developmental regulators or mutant versions of proteins. Conditional gene expression can be achieved at three distinct levels. At the DNA level, inducible site‐specific recombinases allow conditional genome editing. At the RNA level, regulation can be achieved during transcription, using stage‐specific or regulatable promoters, or post‐transcriptionally through alteration of mRNA stability or translation. At the protein level, several systems have been developed for inducible degradation or displacement of a protein of interest. In this review, we provide an overview of current systems for conditional control of gene expression in *Plasmodium* and *Toxoplasma* parasites, highlighting the advantages and limitations of each approach.

## Graphical abstract

Conditional approaches to manipulate gene expression have become instrumental to study the biology of Apicomplexa. Various tools have been developed in *Plasmodium* and *Toxoplasma* parasites, including site‐specific DNA recombinases, regulatable promoters, ribozymes, RNA aptamers and inducible protein degrons.

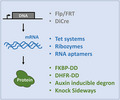

## 1 INTRODUCTION

The phylum Apicomplexa comprises more than 5,000 species, most of which are obligate intracellular parasites, including important pathogens for humans such as *Plasmodium* spp., *Toxoplasma gondii* and *Cryptosporidium* spp. Tremendous progress has been achieved over the past 20 years with the advent of genome sequencing and the availability of powerful bioinformatics interfaces to interrogate genome resources from many species and strains (Harb & Roos, 2015). An additional leap forward comes from the development of a variety of genetic tools to manipulate the genome of apicomplexan parasites. Reverse genetics has become a central approach to explore parasite biology, unravel gene function and identify new targets for therapeutic strategies (Sexton et al., 2019). Genetic manipulation in apicomplexan parasites has been limited until now mainly to *Toxoplasma* and a few species of *Plasmodium* (including *P. falciparum*), but has been greatly facilitated over the past years through the diversification of selection procedures, including flow cytometry assisted parasite sorting, and the advent of genome editing based on CRISPR‐Cas9 (Di Cristina & Carruthers, 2018). In *Toxoplasma*, a major progress has been the development of ΔKu80 strains (Huynh & Carruthers, 2009). The Ku80 protein is involved in DNA repair and is required for the non‐homologous end‐joining pathway that is responsible for the high rate of random integration of transfected DNA in the *T. gondii* genome. In the ΔKu80 *T. gondii* strains, deletion of this gene increases the likelihood of homologous recombination to occur, therefore enhancing the chances of integration to the targeted locus, like in *Plasmodium* parasites. Moreover, together with the CRISPR‐Cas9 technology, this now allows reducing the homology arm length to 30–40 bp for genetic engineering of *T. gondii* parasites, therefore abrogating most of the lengthy cloning steps.

Genetic manipulation requires access to parasite replicative stages to allow the selection of recombinant parasites, that is, asexual blood stages in *Plasmodium* spp. or tachyzoites in *T. gondii*. Some species are easily propagated in culture, such as *P. falciparum*, *P. knowlesi* or *T. gondii*, while others require passage and selection in animals. This is the case for *P. berghei* and *P. yoelii*, two rodent malaria species that can be propagated in laboratory mice and *Cryptosporidium*, which can multiply in immunocompromised mice. Recently, culture methods based on intestinal organoids and air‐liquid interface cultivation systems have been developed that support *C. parvum* growth in vitro, opening new perspectives for the genetic manipulation of this parasite (Heo et al., 2018; Wilke et al., 2019).

Apicomplexan parasites are haploid in stages that can be manipulated, facilitating gene targeting. However, one inherent limitation of genetic manipulation is that genes that are essential for parasite invasion, growth or egress in genetically tractable stages are refractory to direct gene deletion. In this context, conditional approaches are necessary for functional studies targeting essential genes. Genome‐wide screens based on targeted or random mutagenesis showed that around 40% of genes appeared essential in both *P. berghei* and *P. falciparum* asexual blood stages (Bushell et al., 2017; Zhang et al., 2018). Similarly, a genome‐wide CRISPR‐Cas9 screen performed in *T. gondii* revealed that around 40% of genes contribute to parasite fitness during proliferation of tachyzoites in cell cultures (Sidik et al., 2016). Many of these genes represent potential therapeutic targets. Evidence for genetic essentiality is important to validate targets, and conditional approaches are necessary to investigate the function of these genes in other parts of the life cycle. Conditional genome editing can also be applied to non‐essential genes, in particular to avoid adaptation of the parasite that can occur during long selection procedures associated with conventional approaches, and which sometimes results in upregulation of compensatory gene expression masking the biological importance of a gene product. Conditional gene expression can also be used to induce parasite differentiation massively in a population, as exemplified with induced sexual conversion in *Plasmodium* (Filarsky et al., 2018; Kent et al., 2018; Poran et al., 2017) or conversion to bradyzoites in *T. gondii* (Waldman et al., 2020), and can be used to express dominant negative mutants or toxic genes (Kremer et al., 2013).

Three types of conditional approaches can be distinguished, depending on whether gene expression is controlled at the DNA, RNA, or protein level. Conditional genome editing strategies operate at the DNA level and involve inducible recombination. Other strategies act at the RNA level to manipulate the timing and/or level of transcription or alter the stability and/or translation of mRNA. The third class of conditional methods controls the expression of proteins by modifying their stability or their localization. However, all three types of conditional strategies share a same final objective, to achieve temporal control on the expression of a protein of interest, and rely on modification of the parasite genome to introduce control elements in the gene of interest (GOI). While it is possible to conditionally and irreversibly knockout a gene with strategies based on DNA recombination, the other approaches result in a conditional knockdown, which is usually reversible. In the following sections, we summarize the current methods for conditional control of gene expression in *Plasmodium* and *Toxoplasma* parasites and discuss the advantages and limitations of each approach (Table 1).

**TABLE 1:** Advantages and limitations of the different strategies for conditional gene expression in Apicomplexa

Strategies to control gene expression	Systems	Species	Advantages	Limitations	References
Conditional DNA editing	Flp/FRT	*Pb*	High efficiency Applicable in vivo Irreversible knockout	Parental line expressing Flp Restricted to mosquito stages Slow effect on protein	Carvalho et al. (2004), Combe et al. (2009)
	CRE/Lox	*Pf, Pb, Tg*	High efficiency Applicable in vivo Irreversible knockout No stage‐specificity	Parental line expressing DiCre Slow depletion of protein Rapamycin effects	Andenmatten et al. (2012), Collins et al., (2013), Kent et al. (2018)
Regulated gene transcription	Promoter swap	*Pf, Pb*	Simple cloning Applicable in vivo	Stage‐specific regulation	Siden‐Kiamos et al., (2011), Laurentino et al. (2011)
	Tet‐ON	*Tg*	Reversible	Variable efficiency ATc toxicity	Meissner et al. (2001)
	Tet‐OFF	*Pf, Pb, Tg*	High efficiency Applicable in vivo	Parental line expressing TATi ATc toxicity Heterologous promoter Slow effect on protein	Meissner et al. (2002), Pino et al. (2012)
Control of mRNA stability or translation	glmS ribozyme	*Pf*	Simple cloning Reversible Tunable	Variable efficiency GlcN toxicity	Prommana et al. (2013)
	TetR‐DOZI‐aptamer	*Pf*	High efficiency	ATc toxicity	Ganesan et al. (2016)
Control of protein stability	DD domain	*Pf, Tg*	Simple cloning High efficiency Rapid effect on protein Reversible Tunable	Effect of tag Protein access to proteasome Shld−1 toxicity Costly	Armstrong and Goldberg (2007), Herm‐Götz et al. (2007)
	DHFR destabilization domain	*Pf, Cp*	Simple cloning High efficiency Rapid protein depletion Reversible Tunable Applicable in vivo TMP inexpensive	Parental TMP‐resistant line Effect of tag Protein access to proteasome	Muralidharan et al. (2011)
	Auxin‐inducible degron (AID)	*Pf, Pb, Py, Tg*	Simple cloning High efficiency Rapid protein depletion Reversible Tunable Applicable in vivo Auxin inexpensive	Effect of tag Parental line expressing TIR1	Kreidenweiss et al. (2013), Philip and Waters (2015), Brown et al. (2017)
Regulated protein localization	Knock sideways	*Pf*	Rapid inducible protein displacement	Effects of protein tagging Parental line expressing localizer	Birnbaum et al. (2017)

## 2 CONDITIONAL DNA EDITING

Removal of DNA sequences from the genome can be achieved by activating site‐specific recombinases and is the most radical and irreversible method to disrupt gene function. Site‐specific recombination (SSR) can also be used for conditional allelic replacement or recycling of selectable markers. Two SSR systems have been implemented in *Plasmodium* and/or *Toxoplasma*, one based on the yeast Flippase (Flp) and the other on the bacteriophage Cre (Collins et al., 2013; Lacroix et al., 2011). Both recombinases recognize short specific 34‐bp sequences, termed Flippase Recognition Target (FRT) or locus of X‐oxer in P1 (LoxP), respectively. FRT and LoxP sites can be introduced in the parasite genome to flank a target DNA sequence. Flp and Cre will induce DNA recombination between the two FRT or LoxP, respectively, resulting in excision of the flirted/floxed DNA if the two sites are in the same direction, or inversion of the target DNA sequence if the sites are in the opposite orientation. Depending on the position of the FRT/Lox sites, complete or partial gene deletion can be achieved by SSR. Diverse Lox site sequences can be used, which increases the versatility of the system. Modifying the parasite genome to introduce FRT/Lox sites has been greatly facilitated by CRISPR‐Cas9 approaches (Knuepfer et al., 2017). One critical issue with both systems is the positioning of the FRT/Lox sites, which may have detrimental effects on gene expression by disrupting native gene regulatory elements with the introduction of exogenous sequences. To solve this issue, a strategy has been developed in *P. falciparum*, consisting in introducing Lox sites inside the GOI within a natural or artificial intron (Jones et al., 2016). Another option is to flank the 3’ UTR with FRT/Lox sites, to avoid detrimental effects on gene transcription. However, SSR‐mediated removal of the 3’ UTR may not be sufficient to induce gene silencing depending on the GOI for both *Toxoplasma* and *Plasmodium*, as exemplified with CRT in *P. berghei* or SERA5 in *P. falciparum* (Collins et al., 2013; Ecker et al., 2012).

The efficiency of SSR‐based strategies depends on various factors, including the recombination rate, its effects on mRNA and/or protein synthesis in the case of partial gene deletion, as well as mRNA and/or protein stability following DNA excision. All these factors can vary depending on the GOI and the accessibility of the targeted locus. In this regard, the inclusion of a fluorescent marker which expression is switched on (or off) upon DNA recombination can facilitate the monitoring of SSR.

A common limitation of SSR approaches is the need for specific parental parasite lines that express the recombinase. Also, even though DNA excision can be rapid upon activation of the recombinase, SSR is not suitable to achieve rapid depletion of a protein of interest. Recombinase‐based DNA excision is a potent approach as successful gene excision completely prevents gene transcription, however the major challenge is to obtain tight control of the recombinase activity. This has been achieved by expressing the Flp recombinase in a stage‐specific manner, or by using a split version of Cre (DiCre) that can be activated by a small ligand, as detailed below.

### 2.1 The Flp/FRT recombination system

**FIGURE 1:**
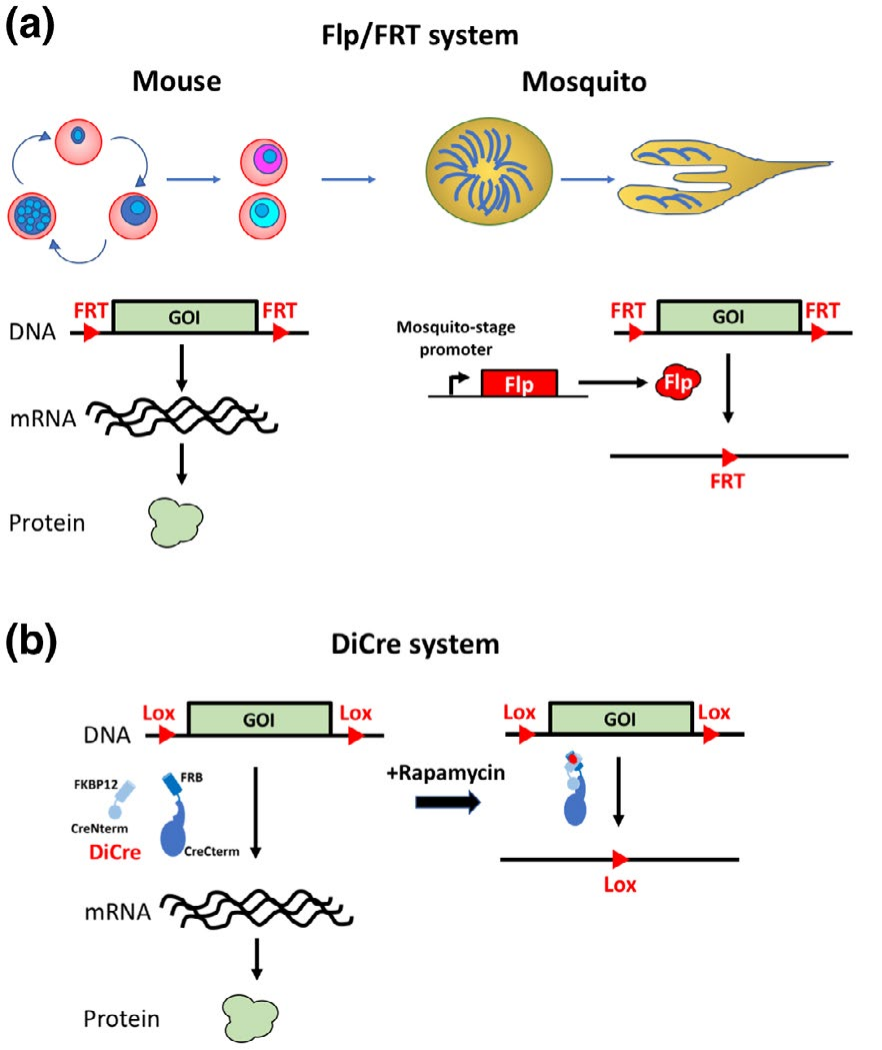
Conditional genome editing. (a) In the Flp/FRT system, stage‐specific expression of the Flp recombinase in mosquito stages results in site‐specific recombination between FRT sites and excision of the flirted target DNA fragment during parasite maturation in the mosquito. (b) In the DiCre system, the Cre recombinase is expressed as two subunits fused to FKBP and FRB, respectively, that dimerize in the presence of rapamycin. Rapamycin‐dependent heterodimerization of the two subunits restores recombinase activity, inducing site‐specific recombination between Lox sites and excision of the floxed target DNA fragment

The Flp/FRT SSR method consists in inserting FRT sites on each side of a target DNA sequence in a parasite line expressing the Flp recombinase via a regulatable or developmental stage‐specific promoter (Figure 1a). The activity of Flp is optimal at 30°C, and the Flp/FRT system was shown to be poorly efficient in *P. falciparum* blood stage cultures (O’Neill et al., 2011). However, the system has been successfully implemented in the *P. berghei* model using stage‐specific promoters active during parasite development in the mosquito to drive expression of the recombinase (Carvalho et al., 2004; Combe et al., 2009; Lacroix et al., 2011). Indeed, Flp activity is maintained at temperatures permissive for parasite development in the mosquito (20–25°C). Several deleter parental parasite lines have been generated in *P. berghei* NK65 and ANKA strains, expressing Flp or its thermolabile variant FlpL under control of *trap* or *uis4* promoter (Lacroix et al., 2011; Panchal et al., 2012). The promoter of *trap* is active in mosquito midgut sporozoites, allowing Flp expression in immature sporozoites, whereas *uis4* is upregulated in salivary gland sporozoites and drives SSR after colonization of the insect salivary glands (Lacroix et al., 2011). In this system, Flp is not expressed in blood stages and the flirted GOI locus is not impacted. After transmission of the parasite to the mosquito, the Flp recombinase is expressed, leading to the excision of flirted DNA. This system thus requires passage through the mosquito to induce SSR, and can be used to confirm the essentiality of a gene and the consequences of gene deletion during pre‐erythrocytic stages (Giovannini et al., 2011). However, it is less suitable for functional studies of essential genes in the blood stages. In addition, SSR occurs late during sporozoite development in the mosquito, with the risk of incomplete depletion due to protein carryover after DNA recombination, as documented for PKG in TRAP/FlpL parasites (Falae et al., 2010; Govindasamy et al., 2016).

### 2.2 Dimerisable Cre

The Cre‐Lox is a highly efficient technology that has been extensively used in many organisms (Hamilton & Abremski, 1984) (Nagy, 2000; Sauer, 1998), including Apicomplexa. Initial attempts to control Cre expression through regulated promoters revealed that leaky transcription combined with the high activity of Cre was sufficient to induce excision of floxed DNA even in the absence of induction (Brecht et al., 1999; O’Neill et al., 2011). An inducible version of Cre, fused to a ligand domain of the estrogen receptor and activated by tamoxifen has been widely used in mammalian systems. However, tamoxifen affects the growth of *P. falciparum* in cultures and *P. berghei* in mice (Weinstock et al., 2019), and in *T. gondii* the Cre fusion with the hormone‐binding domain was found to be constitutively active, limiting the utilization of this conditional system (Brecht et al., 1999). This issue was solved through the use of ligand‐induced activation of a split DiCre (Jullien et al., 2003). The DiCre system relies on the expression of the bacteriophage P1 Cre recombinase in the form of two separate enzymatically inactive polypeptides, a N‐terminal CRE59 fragment and a C‐terminal CRE60 fragment, fused to a different rapamycin‐binding protein (FKBP12 and FRB respectively) (Jullien et al., 2003). Heterodimerization of the two subunits in the presence of rapamycin restores recombinase activity, inducing SSR between Lox sites and excision (or inversion) of the floxed target DNA fragment (Figure 1b). The DiCre system has been implemented in both *Toxoplasma* and *Plasmodium* (Andenmatten et al., 2012; Collins et al., 2013). The system is highly efficient with excision rates approaching 100% in *Plasmodium* (Collins et al., 2013; Fernandes et al., 2020). Upon exposure to rapamycin, Cre activation occurs rapidly, with complete DNA recombination achieved in a few hours (Kent et al., 2018). The DiCre SSR system has now been extensively used in *P. falciparum* for conditional knockout of a variety of GOI, including those encoding essential invasion proteins or signaling components (Flueck et al., 2019; Perrin et al., 2018; Tibúrcio et al., 2019; Wilde et al., 2019). DiCre has also been implemented in the widely used rodent malaria model parasite *P. berghei*, providing a specific, rapid, robust and tunable conditional system (Fernandes et al., 2020; Kent et al., 2018). It has also been used for allelic replacement, for example to express mutated versions of proteins (Bui et al., 2019), or for excision of selectable markers (Fernandes et al., 2020). Induction of sexual conversion has been achieved in *Plasmodium* through DiCre‐based inversion of a promoter sequence to drive expression of the master regulator AP2‐G (Kent et al., 2018; Llorà‐Batlle et al., 2020). Of interest, the DiCre approach was shown to be suitable to investigate the function of essential *Plasmodium* genes in mosquito and pre‐erythrocytic stages. Rapamycin can be administered immediately before parasite transmission or directly to the mosquitoes, allowing SSR activation in mosquito stages (Fernandes et al., 2020; Tibúrcio et al., 2019). The DiCre system thus appears as a highly efficient and versatile system that allows targeting genes at multiple stages of development. The DiCre system has also been used in *T. gondii* to delete essential genes (Andenmatten et al., 2012), although to a lesser extent as compared to *P. falciparum*. This is mainly due to the weak excision performance of the original parental strain expressing DiCre (Venugopal et al., 2017) and the complexity of cloning steps when trying to target large genes. However, a new parental strain, expressing both DiCre subunits in the same transcriptional subunit, greatly enhances the excision performance (Hunt et al., 2019). DiCre was also used to conditionally insert U1 recognition sites in the terminal exon of GOI in *T. gondii*, resulting in efficient silencing by the spliceosomal component U1snRNP (Hammoudi et al., 2018; Pieperhoff et al., 2015).

## 3 REGULATED GENE TRANSCRIPTION

**FIGURE 2:**
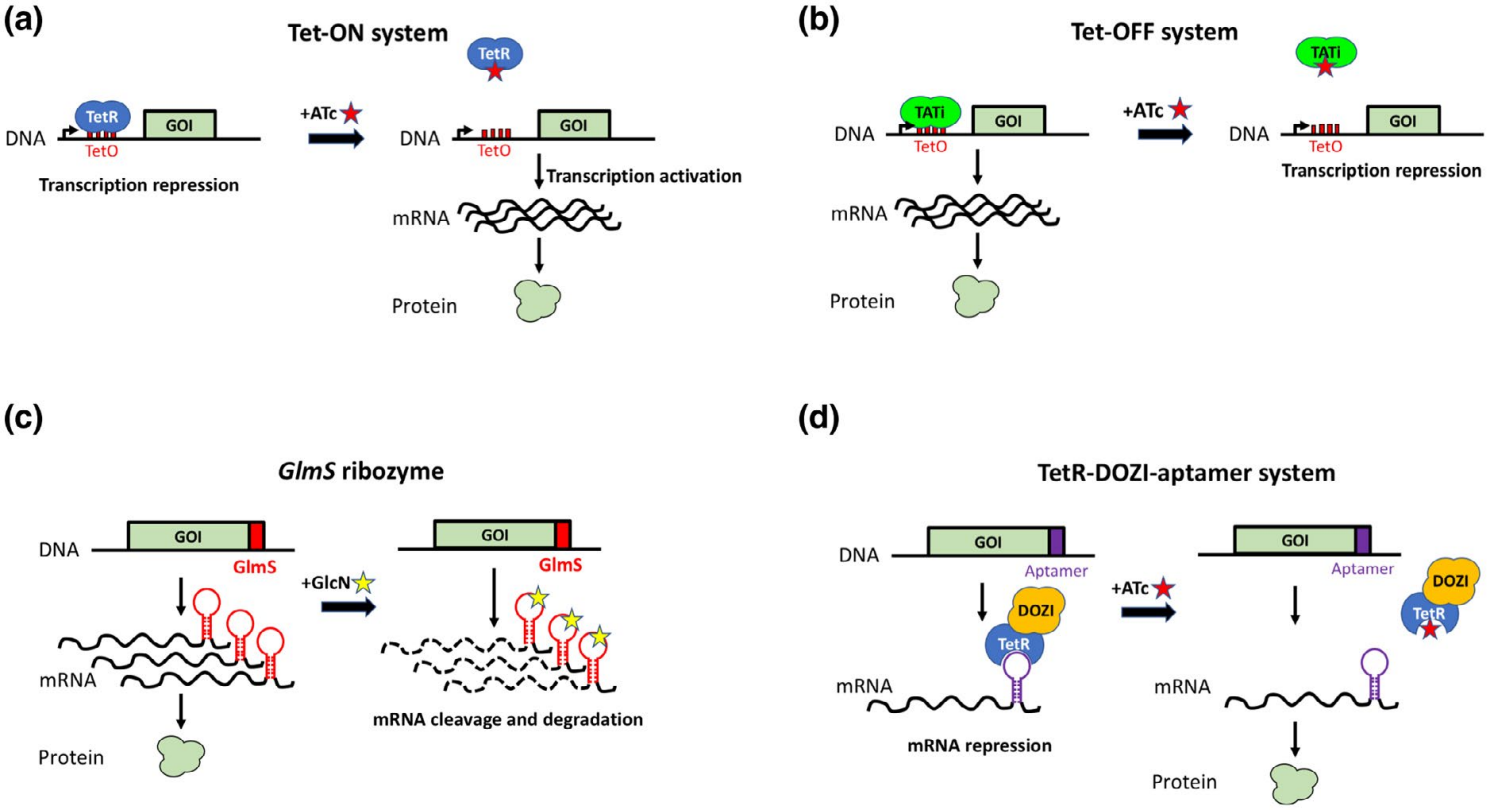
Conditional control of gene expression at the RNA level. (a) The Tet‐ON method is based on the TetR repressor that inhibits transcription through binding to tetO elements placed in the promoter region of a gene of interest (GOI). In the presence of anhydrotetracycline (ATc), TetR is displaced from the promoter, allowing gene transcription. (b) In the Tet‐OFF system, TetR fused to a transactivator domain (TATi) binds to tetO elements placed in a minimal promoter upstream of the GOI, allowing transcription. In the presence of ATc, the TeR‐TATi is displaced, resulting in transcription inhibition. (c) *GlmS* ribozyme is a catalytic RNA element that can be introduced in the 3’ UTR of a GOI. *GlmS* is activated in the presence of glucosamine (GlcN), resulting in mRNA cleavage and degradation. (d) In the TetR‐DOZI‐aptamer strategy, a TetR‐DOZI fusion protein binds to aptamer elements introduced in the 3’ UTR of a GOI, resulting in the relocalization of the mRNA to P bodies and translational repression. In the presence of ATc, TetR‐DOZI is displaced and the mRNA can be translated

Apicomplexa display transcription profiles that are tightly regulated during their life cycle. The characterization of stage‐specific promoters has enabled strategies to control gene expression through simple promoter exchange (Figure 2a). This strategy has been applied in *P. berghei* to knockdown genes in gametocytes or ookinetes, by exchanging the endogenous promoter with a promoter that is active in asexual blood stages but not transmission stages (Laurentino et al., 2011; Siden‐Kiamos et al., 2011), or for stage‐specific knock‐down of rhoptry genes in sporozoites by replacing their endogenous promoter by that of *MSP1* or *MSP5* genes (Bantuchai et al., 2019; Ishino et al., 2019; Nozaki et al., 2020). While these approaches do not require the use of a specific parental line, the heterologous promoter may lead to overexpression and/or inappropriate expression timing, with potentially deleterious effects or protein mislocalization. In addition, simple promoter exchange approaches are limited to stage‐specific alteration of gene expression.

### 3.1 Tetracyclin‐regulated promoters

To achieve tunable control of gene expression, more sophisticated strategies have been developed in *Toxoplasma* and *Plasmodium*, based on tetracycline (Tet)‐regulatable promoters (Meissner et al., 2001, 2005; Meissner et al., 2002). Two versions of Tet‐dependent gene regulation are available, allowing either conditional activation (Tet‐On) or repression (Tet‐Off) of transcription of the GOI. Tet‐based methods use anhydrotetracyclin (ATc) instead of tetracycline or doxycycline, as it is less toxic to the parasite (Meissner et al., 2001).

The Tet‐On strategy is based on the tetracycline resistance operon of *E. coli* Tn10 transposon, and requires two components, tetracyclin operator (tetO) sequences introduced upstream of the transcriptional start site in the promoter of the GOI, and the Tetracyclin repressor (TetR) (Meissner et al., 2001). The binding of TetR on tetO sequences interferes with the recruitment of the transcription machinery and blocks transcription of the gene. In the presence of tetracycline derivatives, TetR dissociates from tetO sequences, allowing gene transcription (Figure 2b). In *Toxoplasma*, using a bulkier version of TetR fused to YFP increased the regulation efficiency (van Poppel et al., 2006). Introduction of multiple tetO elements may also impair promoter activity, although a suitable promoter for tetO integration was identified (van Poppel et al., 2006). The Tet‐on system suffers from several limitations, including residual promoter activity in the absence of ATc, and the need to maintain parasite growth under constant exposure to ATc, which can be toxic, in order to achieve conditional knockdown of essential genes. However, it has been used as an approach to fine‐tune the controlled overexpression of proteins (Etheridge et al., 2014).

In the Tet‐Off system, tetracycline derivatives are used to conditionally repress the expression of the GOI. It relies on a transactivator composed of TetR fused to a transactivator domain that is able to induce the recruitment of the transcription machinery (Meissner et al., 2002). In *T. gondii*, the transactivator domain is a synthetic sequence that was selected for its ability to induce gene expression (Meissner et al., 2002). This hybrid polypeptide can bind to tetO sequences fused to a minimal promoter placed upstream of the GOI, thereby activating gene expression. In the presence of ATc, binding of the transactivator to tetO sequences is impaired and transcription of the GOI is switched off (Figure 2c). Minimal promoters used in *Toxoplasma* are derived from the gene promoters of TgSAG1 or TgSAG4 (Meissner et al., 2001). More recently, a GRA2‐derived minimal promoter was shown to allow more robust regulation (Sharifpour et al., 2021). The Tet‐off system has been first developed in *Toxoplasma* using the transactivator TATi‐1 (Trans‐Activator Trap identified), which was selected through a genetic screen based on random integration of TetR in *T. gondii* (Meissner et al., 2002) and was later shown to work as well in *P. falciparum* (Meissner et al., 2005). A parasite strain expressing the TATi‐1 transactivator in a ΔKu80 background was created to facilitate genetic engineering of endogenous promoter sequences via homologous recombination (Sheiner et al., 2011). The TATi‐1 system has now been extensively used in *Toxoplasma* for conditional knockdowns or various GOI. An alternative strategy has been developed in *P. berghei*, based on activating domains from Apicomplexa AP2 transcription factors as TetR‐activating domains (Pino et al., 2012). However, this system was only scarcely used since then.

The Tet‐off system has some limitations. Exchanging the endogenous promoter can result in alterations of the level or kinetics of expression of the GOI (Lesage et al., 2018; Mital et al., 2005), with potential detrimental effects even in the absence of ATc. Moreover, promoter replacement proved impossible for number of GOI when the level or kinetics of expression was essential for the activity of the gene product. To mitigate this drawback, the TATi‐1 transactivator can be placed under the control of the promoter of the targeted gene (Lamarque et al., 2014). Complete protein knockdown sometimes necessitates long parasite exposure to ATc, depending on the protein turn over, with the risk of accumulating secondary phenotypes over time that render data interpretation difficult. However, the system has proved easy to use in vivo in mouse models with the limitation that the TATi strain was shown to have reduced virulence compared to the parental strain (Lesage et al., 2018). This type of approach has been used in *P. berghei* but not *P. falciparum* (Chisholm et al., 2016). Both the Tet‐on and Tet‐off strategies require a specific parental parasite line, expressing the TetR or the transactivator, respectively. In both cases, regulation is reversible but the system inherent inertia (time needed to completely abrogate protein expression) makes it difficult to use in a reversible manner.

## 4 CONTROL OF mRNA STABILITY OR TRANSLATION

In the absence of a functional RNAi pathway in *Plasmodium* and *Toxoplasma*, alternative strategies have been developed to control gene expression post‐transcriptionally at the mRNA level, based on inducible ribozymes or TetR‐aptamer systems.

### 4.1 Ribozymes

Ribozymes are self‐cleaving RNA elements that retain autocatalytic activity when placed in various RNA contexts, and which can be engineered to respond to ligands. Initial proof‐of‐concept studies used the Sm1 hammerhead ribozyme of *Schistosoma mansoni* to regulate gene expression in *Toxoplasma* and *P. falciparum* (Agop‐Nersesian et al., 2008), but the absence of specific and non‐toxic inhibitors has limited its use. In contrast, the Glucosamine‐inducible *glmS* ribozyme has emerged as a potent system for inducible knockdown of gene expression in *P. falciparum* (Prommana et al., 2013). The *glmS* ribozyme is a natural ribozyme originating from Gram‐positive bacteria, and requires glucosamine‐6‐phosphate for catalytic activity, providing a simple and tunable platform for inducible knockdown strategies. In this system, the ribozyme sequence is introduced in the 3’UTR of a GOI, leading to the expression of a chimeric mRNA containing the ribozyme element. *GlmS* self‐cleavage can be induced by the addition of glucosamine (GlcN) in the culture medium, leading to instability and degradation of the chimeric mRNA (Figure 2d). Regulation is tunable as the level of self‐cleavage can be controlled by varying the concentration of GlcN. Also, an inactive version of the *glmS* ribozyme, M9, which contains a single point mutation that abrogates autocatalytic activity, provides a suitable control in knockdown experiments (Prommana et al., 2013). Another advantage of this system is that it requires no additional genetic element since all the information is contained within the autocatalytic RNA. In particular, the endogenous promoter of the GOI can be preserved to avoid interference with transcription. However, insertion of the *glmS* element can alter gene expression and may cause over‐expression due to the stabilization of the transcripts (Jankowska‐Döllken et al., 2019). In addition, the level of knockdown varies depending on the GOI and the protein turnover, with lower efficacy for proteins that are highly expressed or with low turnover. While *glmS* is rather simple to implement, only requiring modest molecular cloning and addition of a molecule in the medium to induce knockdown, glucosamine can be toxic to the parasite at high concentrations. The *glmS* has been used with success to target various GOI in *P. falciparum* (Aroonsri et al., 2019; Ghosh et al., 2018; Liu et al., 2020; Sheokand et al., 2021), and also operates in *P. berghei* (Aroonsri et al., 2016).

### 4.2 TetR‐aptamers

RNA aptamer systems allow synthetic translational control via ligand‐controlled RNA‐protein modules that can be introduced in native gene contexts. One such system has been developed in *P. falciparum* for tetracyclin‐controlled gene regulation at the mRNA level. The approach is based on genetically encoded TetR‐binding RNA aptamers introduced in the 5’ UTR of a GOI (Goldfless et al., 2014). In this system, in the absence of ATc, TetR binds to the aptamer and prevents translation of the mRNA. When ATc is added, TetR dissociates from the aptamer, allowing mRNA translation and protein synthesis (Figure 2e). This system was shown to be functional in the context of several native and engineered promoters, allowing rapid induction of gene expression.

The RNA aptamer approach has been further refined in *P. falciparum* by fusing the TetR repressor to DOZI, a protein involved in translational repression through mRNA sequestration in P‐bodies (Figure 2f; Ganesan et al., 2016). This approach resulted in higher regulation efficiency as compared to TetR, especially when an array of 10 aptamers was introduced in the 3’ UTR in combination with a single aptamer element placed in the 5’ UTR of the target gene. Similar results were also obtained with TetR fused to another post‐transcriptional effector, CITH. Both TetR‐DOZI and TetR‐CITH provided substantially improved translational regulation over TetR alone (Ganesan et al., 2016). This system has been successfully used in *P. falciparum* for conditional knockdown of various genes (Gupta et al., 2020; Ling et al., 2020; Polino et al., 2020). One potential issue is the loss of one or several aptamer copies following recombination in the parasite genome, leading to a loss of knockdown efficiency. This was recently solved by redesigning the aptamer array to minimize recombination while preserving the control elements (Rajaram et al., 2020).

## 5 CONTROL OF PROTEIN STABILITY

Conditional approaches acting directly at the protein level rely on genetically encoded protein elements that are fused to a protein of interest, which can then be directed to degradation pathways under specific conditions. These approaches allow fast protein knockdown and are thus more suitable than DNA‐ or RNA‐based strategies to study rapid cellular processes. Three different systems have been developed in *Plasmodium* and *Toxoplasma* parasites, the FKBP‐DD and DDD systems, which both rely on destabilization domains causing protein degradation in the absence of a stabilizing agent, and the auxin‐inducible degron system (AID), which allows inducible protein depletion upon addition of auxin. A common limitation to all three methods is the need to incorporate a specific tag fused to the protein of interest, which may alter its function.

### 5.1 The FKBP‐DD system

**FIGURE 3:**
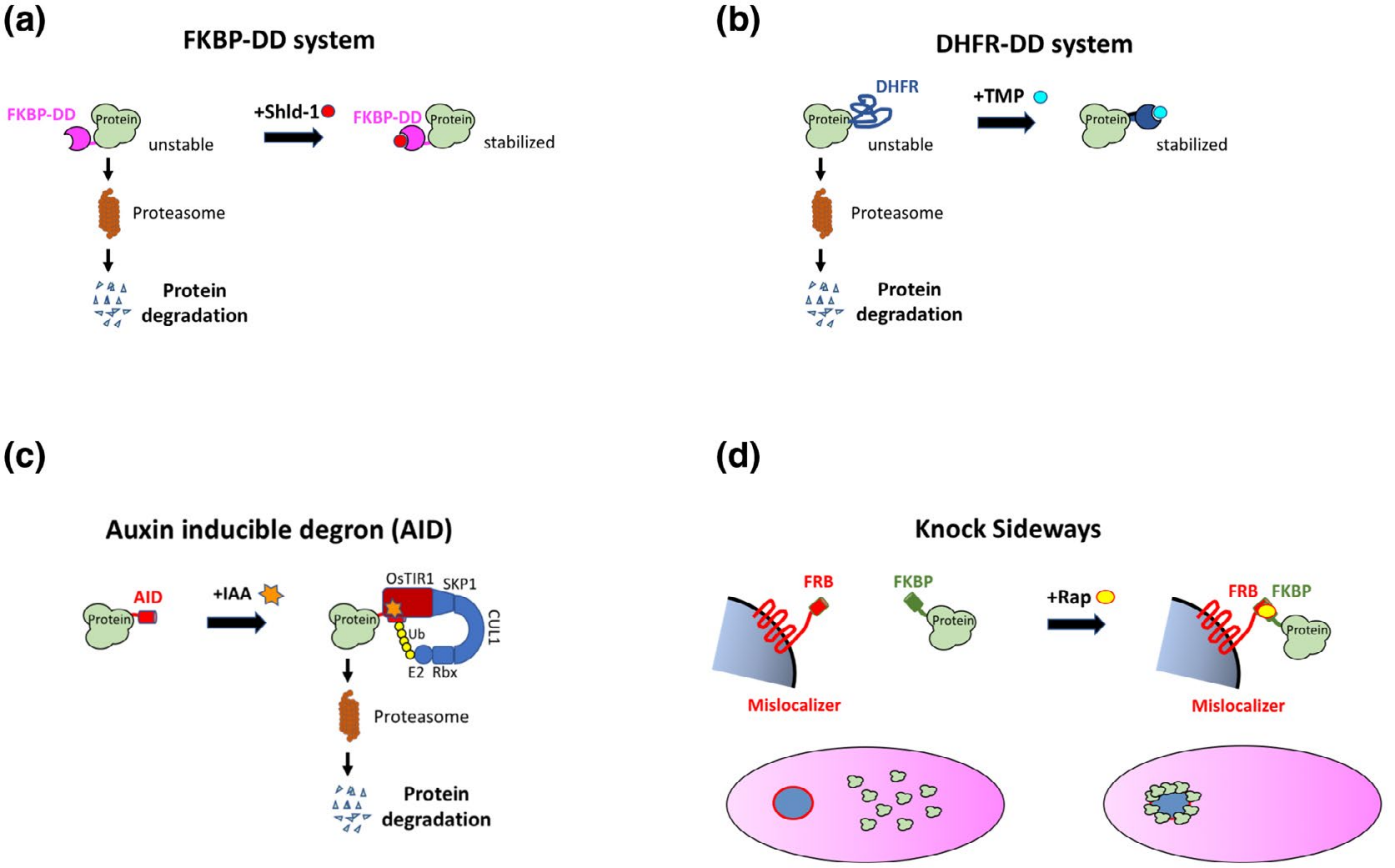
Protein‐based conditional strategies. (a) In the FKBP‐DD system, a FKBP degradation domain fused to a protein of interest causes protein degradation by the proteasome. In the presence of the Shield‐1 (Shld‐1) compound, the DD domain is stabilized and the protein escapes degradation. (b) In the DDD system, a DHFR degradation domain appended to a protein of interest is responsible for proteasome‐dependent protein degradation. In the presence of trimethoprim (TMP) the DHFR DD domain is stabilized and the protein escapes degradation. (c) In the auxin inducible degron (AID) method, an AID tag is introduced in a protein of interest. In the presence of indole‐3‐acetic acid (IAA), genetically encoded TIR1 binds to the AID tag and recruits a E3 ubiquitin ligase complex (Skp, Cullin, F‐box, Rbx), resulting in protein ubiquitination and degradation by the proteasome. (d) The knock sideways strategy relies on two components, a FKBP tag appended to a protein of interest, and a mislocalizer protein fused to FRB and containing targeting motifs to a specific cellular compartment. In the presence of rapamycin, FKBP binds to FRB, resulting in sequestration of the protein of interest in the mislocalizer compartment

The FKBP‐DD system is based on the destabilization domain of the FK506‐binding protein FKBP12, which contains destabilizing mutations that induce misfolding in the absence of the rapamycin‐derived ligand Shield (Shld‐1) (Banaszynski et al., 2006). The DD system has been successfully adapted to modulate protein expression in *Toxoplasma* and *Plasmodium* (Armstrong & Goldberg, 2007; Herm‐Götz et al., 2007). When fused at the N‐ or C‐terminus of a target protein, the FKBP‐DD domain interferes with protein stability, resulting in protein degradation by the proteasome. In the presence of Shld‐1, the DD domain is stabilized and the protein escapes degradation (Figure 3a). One advantage of the DD system is that protein depletion is rapid and reversible. In *Toxoplasma*, a YFP‐DD fluorescent reporter could be fully induced within 90 min upon addition of Shld‐1, and extinguished in about 5 hr following removal of Shld‐1 (Herm‐Götz et al., 2007). The DD method is thus well‐suited to interfere with proteins involved in fast processes such as trafficking or signaling pathways. It is also tunable by varying the concentration of Shld‐1. However, Shld‐1 is costly and can be toxic when administered for long periods or at high concentrations. Conditional knockdown of essential proteins is possible yet implies the continuous exposure of the parasite to Shld‐1 during selection of recombinant parasites. On the opposite, the DD system is particularly well suited for the expression of dominant negative mutants or toxic genes and was used to investigate the role of Rab proteins in *T. gondii* (Kremer et al., 2013). Nevertheless, Shld‐1 can impact parasite growth, which can be confounding in the phenotypical analysis. The poor bioavailability of Shld‐1 in vivo is a limitation for its usage in mice. The DD domain works optimally when positioned at the N‐terminus of the target protein, which renders the system less appropriate for secreted proteins (Limenitakis & Soldati‐Favre, 2011). Degradation of the target protein requires access to the cytoplasmic proteasome, implying that the DD‐mediated regulation works optimally with cytoplasmic proteins but may not be suited for secreted proteins, although efficient knockdown of the *P. falciparum* vacuolar protein falcipain‐2 could be achieved with the DD system (Armstrong & Goldberg, 2007). The efficacy of the DD‐mediated regulation may thus vary depending on the GOI. Also, introduction of the 12‐kDa DD domain may impair protein function, even when stabilized by the ligand, as exemplified with MyoA (Herm‐Götz et al., 2007). The DD system does not rely on specific promoter elements, and thus the endogenous promoter can be used, avoiding complications resulting from inappropriate expression level or timing. The DD domain has now been extensively used for overexpression of dominant negative copy of genes in *T. gondii* (Agop‐Nersesian et al., 2010; Breinich et al., 2009; Daher et al., 2010; van Dooren et al., 2009; Jia et al., 2017) and to a lesser extent *P. falciparum* (Azevedo et al., 2013). In *P. falciparum*, the DD has been used for conditional knockdown of a variety of essential genes (Armstrong & Goldberg, 2007; Blomqvist et al., 2020; Brancucci et al., 2014; Josling et al., 2015; Kumar et al., 2017; Robbins et al., 2017). The DD system was also used for conditional expression of Cas9 in *T. gondii* (Serpeloni et al., 2016) or AP2G in *P. falciparum* (Poran et al., 2017).

### 5.2 The DHFR destabilization domain system

The DDD system is based on a mutated *E. coli* dihydrofolate reductase (DHFR) destabilization domain, which can be stabilized by the folate analogue trimethoprim (TMP) (Iwamoto et al., 2010) (Figure 3b). This method has been implemented in *P. falciparum* (Muralidharan et al., 2011). TMP is an inexpensive compound with antimalarial activity, therefore the DDD system necessitates working with parasites containing a human DHFR cassette that confers resistance to TMP. Similar to the DD system, tagging with the DDD can impact the protein function, even in the presence of TMP. The selection of transgenic parasites for knockdown of essential proteins requires the constant supply of TMP during the selection procedure. TMP has good pharmacological properties and can be used in mice, thus the DDD system could be adapted to *P. berghei*. Interestingly, the DDD tagging appears to be a relevant approach to study chaperones in *P. falciparum*, where binding of the DDD‐tagged chaperone to the unfolded domain prevents interactions with client proteins (Beck et al., 2014; Muralidharan et al., 2012). The DDD approach has been recently adapted to *Cryptosporidium* (Choudhary et al., 2020).

### 5.3 The auxin‐inducible degron system

Another expanding method to conditionally control protein levels is the plant‐derived auxin‐inducible degron (AID) system (Nishimura et al., 2009). In plants, the hormone auxin induces rapid degradation of AUX/IAA transcription repressors by a SCF (Skp1‐Cullin‐F‐box protein) E3 ubiquitin ligase complex comprising the TIR1 protein. While TIR1 is a plant‐specific protein, the SCF degradation pathway is conserved in other eukaryotes, including Apicomplexa, where an auxin response system can be activated upon TIR1 transgenic expression. In the AID method, an auxin‐responsive element (degron) is fused to a protein of interest, in a parental parasite line expressing TIR1. Upon addition of the small molecule auxin (indole‐3‐acetic acid, IAA), the AID‐tagged protein is targeted to the SCF E3 ubiquitin ligase by TIR1 via its F‐box domain, resulting in degradation of the ubiquitinated protein by the proteasome (Figure 3c). This approach has been successfully implemented in *P. falciparum* (Kreidenweiss et al., 2013), *P. berghei* (Philip & Waters, 2015), *P. yoelii* (Liu et al., 2021) and *T. gondii* (Brown et al., 2017; Brown et al., 2018), albeit with contrasting efficiencies (Zeeshan et al., 2021). The AID approach allows rapid (within 30 min) degradation of the tagged protein, and is reversible and in theory tunable by varying the auxin concentrations. One advantage is the small size of the AID tag in its mini version (68 amino acids) (Brown et al., 2017), reducing the risk of interference of the tag with protein function. The AID tag can be inserted at the 3’ end of the gene coding sequence, and therefore does not interfere with the endogenous promoter activity. The AID method works well with cytosolic or nuclear proteins, but also on integral membrane protein of the alveoli (Harding et al., 2019). A major advantage of this system is the rapid degradation of the targeted protein and its reversibility. Indeed, several studies have shown that phenotypes caused by the depletion of a targeted protein can be reversed by simply washing out auxin from the culture media (Hu et al., 2020; Khelifa et al., 2021). The AID system can also be used in vivo in mice, although in this case auxin needs to be provided by oral gavage and in the drinking water (Brown & Sibley, 2018).

Another system relies on a ligand‐induced degradation (LID) domain that can be appended to a protein of interest, and which consists of the FKBP protein fused to a small degron (Bonger et al., 2011). In the absence of Shld‐1 the degron is bound to the FKBP moiety and the protein is stable. When Shld‐1 is added, it binds to FKBP and displaces the degron, which is responsible for rapid degradation of the LID domain and the fused partner protein. This system has been recently used in *P. falciparum* to knockdown the PfSWIB protein (Wang & Zhang, 2020).

## 6 REGULATED PROTEIN LOCALIZATION

One strategy to interfere with the function of a protein is to alter its localization. Such an approach, termed knock sideways (KS), has been developed in *P. falciparum* using the rapamycin‐dimerizable domains FKBP and FRB (Birnbaum et al., 2017). The protein of interest is tagged with FKBP in a parasite line that expresses a mislocalizer protein, which consists of the FRB domain fused to a targeting signal for localization in a specific compartment (nucleus or plasma membrane for example). Addition of rapamycin allows FKBP‐FRB interaction and induces relocalization of the FKBP‐tagged protein to the target site and depletion from its normal site of action (Figure 3d). One limitation of this system, similar to the protein degradation methods described above, is that the mislocalizer protein must be accessible to the FKBP‐tagged protein for KS to be efficient. In addition, the fusion with the FKBP tag may alter protein function, irrespective of rapamycin exposure. Theoretically, mislocalization of the FRB‐tagged protein in the presence of rapamycin may also be a confounding event and proteins may interact with non‐natural interactors in the newly targeted compartment.

A strategy based on conditional localization domains (CLD) has been designed to specifically control apicoplast protein trafficking. Export of nuclear‐encoded apicoplast proteins relies on a N‐terminal bipartite signal comprised of a signal peptide and a transit peptide. The transit peptide must be unstructured to allow efficient trafficking to the apicoplast. Roberts et al developed a system where a CLD derived from FKBP is appended to a protein of interest. In the absence of the ligand Shld‐1, the CLD is unstructured and the protein traffics normally to the apicoplast. In the presence of Shld‐1, the CLD is stabilized, causing the tagged protein to be secreted from the parasite. This approach was used in *P. falciparum* to conditionally localize the biotin ligase HCS1 to the apicoplast in asexual blood stages (Roberts et al., 2019).

## 7 METABOLIC RESCUE

Conditional disruption of genes encoding essential apicoplast proteins has been achieved through chemical rescue in *P. falciparum* (Yeh & DeRisi, 2011). This method exploits the fact that the only essential function of the apicoplast during the asexual blood stages is to synthesize isoprenoid precursors. The apicoplast becomes dispensable when the isoprenoid precursor isopentenyl pyrophosphate (IPP) is provided in the culture medium, permitting conditional disruption of genes that are essential for the biogenesis or maintenance of the plastid (Yeh & DeRisi, 2011). More recently, a transgenic line expressing a functional heterologous mevalonate pathway was engineered in *P. falciparum*, allowing synthesis of the isoprenoid precursors in parasites supplemented with mevalonate, providing another platform for conditional disruption of apicoplast genes (Swift et al., 2020).

## 8 CONCLUSION AND FUTURE PROSPECTS

The toolbox for conditional gene regulation in Apicomplexa has now expanded considerably over the past years. Many methods are available for conditional gene expression, each with their advantages and limitations. There is not a single universal approach permitting rapid, efficient, tunable and reversible regulation of any GOI, therefore the choice of the method should be made after careful consideration of the context. When complete and irreversible gene knockout is desired, SSR‐based conditional genome editing is probably the best option. In this respect, the DiCre strategy has been shown to work on virtually all the life cycle stages of *Plasmodium*, which is not the case for other approaches. In general, low turnover of mRNA and/or protein increases the time needed to achieve sufficient depletion with DNA and RNA‐based methods. Therefore, protein degradation approaches are the best suited for rapid depletion of proteins, provided that the localization of the protein of interest allows access to the degradation pathways. When possible, several approaches should be attempted, and different strategies can be combined to increase the efficiency of gene expression control. As an example, a dual regulation strategy combining ATc‐mediated transcription suppression and FKBP‐DD‐mediated protein degradation was used for more robust and stable knockdown of Centrin 2 in *Toxoplasma* (Leung et al., 2019).

One can anticipate that the toolbox will expand further in the next coming years with implementation of techniques developed in other organisms, including CRISPR interference (CRISPRi) strategies, which are based on a catalytically inactive dead Cas9 enzyme (dCas9). Targeting of the dCas9 using specific gRNA to the region upstream of a GOI can inhibit transcription initiation or elongation, resulting in reduction in gene expression. CRISPRi strategies have already been implemented in *P. falciparum* and *P. yoelii* to inhibit the expression of specific loci (Barcons‐Simon et al., 2020; Baumgarten et al., 2019; Walker & Lindner, 2019). The dCas9 can also be fused with epigenetic effector domains to activate or repress transcription via hyperacetylation or hypoacetylation of chromatin, respectively (Xiao et al., 2019). Conditional CRISPR‐based approaches can now be envisaged based on inducible dCas9 systems to knockdown essential genes. In this respect, a major progress has been the development of the ribozyme‐guide‐ribozyme (RGR) method, which now allows expressing single guide RNA (sgRNA) from RNA polymerase II (instead of RNA polymerase III) promoters (Walker & Lindner, 2019). This opens the possibility to use stage‐specific or regulatable promoters to control the expression of sgRNA for CRISPR‐RGR conditional control of gene expression. Alternatively, methods have been developed to control Cas9 activity, through chemical activation of a split Cas9 (Zetsche et al., 2015) or based on photoactivable split Cas9 (Yu et al., 2020). Optogenetics techniques in general are developing in many fields, including parasitology. As an example, an optogenetic approach based on the photoactivated adenylate cyclase from the lithotropic bacterium *Beggiatoa* allowed rapid spatiotemporal control of cAMP levels in *Toxoplasma* (Hartmann et al., 2013).

Finally, as illustrated in this review, conditional gene manipulation in Apicomplexa has been restricted so far to laboratory strains of *P. falciparum*, *P. berghei*, and *T. gondii*. The availability of whole genome sequence data and progress in genome editing now open new avenues to envisage extending these approaches to a wider range of parasite strains and species that are amenable to genetic manipulation, including *Cryptosporidium* and the simian malaria parasites *P. knowlesi* and *P. cynomolgi*. In the post‐genomic era, and owing to the progress in genome editing methods, one can expect that conditional genetic approaches will play an ever‐growing role in the study of Apicomplexa biology, to unravel gene function and ultimately identify novel therapeutic targets.

## CONFLICT OF INTEREST

No conflict of interest declared.

## Data Availability

Data sharing is not applicable to this article as no new data were created or analyzed in this study.
